# Endoscopic submucosal dissection with dental floss traction for the treatment of a superficial tumor in the horizontal part of the duodenum

**DOI:** 10.1055/a-2307-6039

**Published:** 2024-05-07

**Authors:** Xiaolong Xian, Silin Huang, Suhuan Liao, Jun Cai, Guang Yang, Bo Li, Rui Feng

**Affiliations:** 1 Department of Gastroenterology, South China Hospital, Medical School, Shenzhen University, Shenzhen, China

A 55-year-old man, during a routine screening gastroscopic examination, was found to be harboring an incipient neoplasm on the horizontal part of the duodenum. The lesion measured approximately 40×35 mm. Its surface morphology exhibited irregularity and a villous-like appearance under linked color imaging or blue light imaging, with uneven distribution and marginalization of white opaque substance. Abdominal enhanced computed tomography showed no lymphatic or organ metastasis ( Fig. 1 ). The patient was admitted as an inpatient and underwent endoscopic submucosal dissection (ESD) under general anesthesia ( Video 1 ). Significant challenges were encountered during this procedure, specifically during the circumferential mucosal incision, and particularly on the anal side of the lesion. To overcome the difficulty, we employed a colonoscope (EC-L600ZP7; Fujifilm, Tokyo, Japan) and applied abdominal pressure, which enhanced endoscope stability and allowed the circumferential incision to be successfully completed. We then used clips and dental floss for traction on the oral side of the lesion. This improved visualization of the submucosal layer and facilitated continuous pulling towards the oral side, aiding the approach of the endoscope’s tip. We transitioned to a gastroscope (EG-601WR; Fujifilm, Tokyo, Japan) to complete dissection in the submucosal layer. The procedure was completed in 285 minutes ( Fig. 2 ). After surgery, no complications such as perforation, hemorrhage, or pyrexia occurred. Histopathological analysis showed complete excision of the villous tubular adenoma, with R0 resection achieved ( Fig. 3 ).

**Fig. 1:**
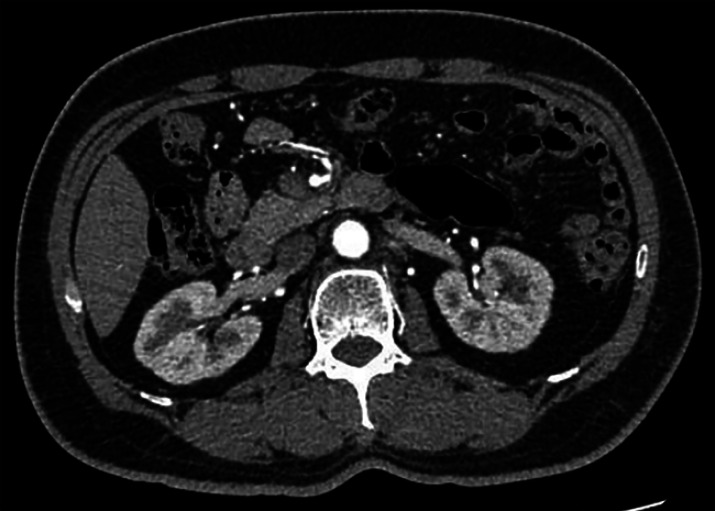
Enhanced computed tomography showing thickening of the duodenal horizontal wall.

**Fig. 2:**
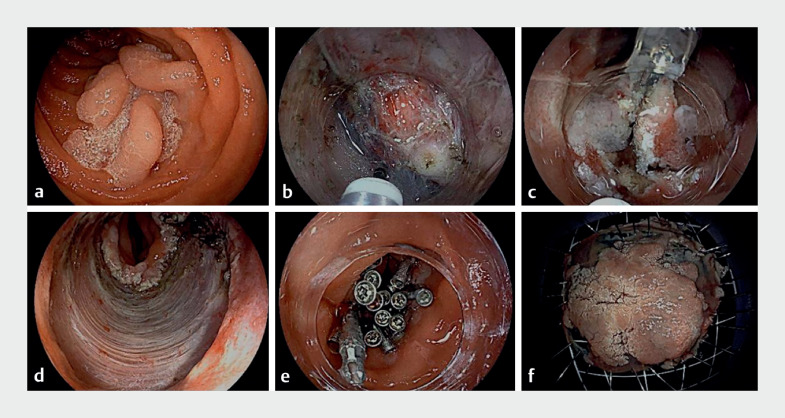
Endoscopic submucosal dissection. **a** Superficial tumor in the horizontal part of the duodenum. **b** Submucosal dissection. **c** Dental floss traction. **d** Postoperative defect. **e** Closure of the defect with metal clips. **f** Resected tumor.

**Fig. 3:**
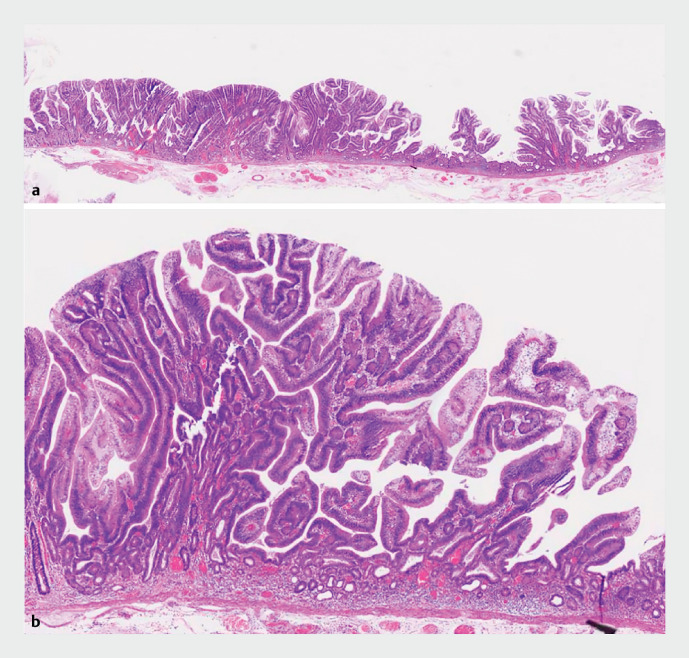
Histopathological analysis demonstrating villous tubular adenoma. **a** Low-power field. **b** High-power field.

Endoscopic submucosal dissection with dental floss traction for the treatment of a superficial tumor in the horizontal part of the duodenum in a 55-year-old man. WLE, white-light endoscopy; LCI, linked color imaging; BLI, blue light imaging.Video 1

Given the rarity of duodenal tumors, reports on superficial tumors within the horizontal part of duodenum are scarce 1 . The duodenum’s distinctive anatomical characteristics, with a small lumen and a C-shaped cavity, give rise to challenges in carrying out ESD 2 . Instances of dental floss traction in ESD interventions for superficial duodenal tumors have been documented 3 .

To our knowledge, this is the first report of the employment of both a gastroscope and a colonoscope, together with the application of dental floss traction, to address the challenges encountered during ESD for a superficial tumor situated in the horizontal segment of the duodenum.

Endoscopy_UCTN_Code_TTT_1AO_2AG_3AD

## Notes

Endoscopy E-Videos https://eref.thieme.de/e-videos
*E-Videos* is an open access online section of the journal *Endoscopy* , reporting on interesting cases and new techniques in gastroenterological endoscopy. All papers include a high-quality video and are published with a Creative Commons CC-BY license. Endoscopy E-Videos qualify for HINARI discounts and waivers and eligibility is automatically checked during the submission process. We grant 100% waivers to articles whose corresponding authors are based in Group A countries and 50% waivers to those who are based in Group B countries as classified by Research4Life (see: https://www.research4life.org/access/eligibility/ ). This section has its own submission website at https://mc.manuscriptcentral.com/e-videos .

## References

[1] Alkhatib AA Sporadic nonampullary tubular adenoma of the duodenum: prevalence and patients’ characteristics Turk J Gastroenterol 2019 30 112 113 10.5152/tjg.2018.17823 30429109 PMC6389308

[2] Kuroki K Sanomura Y Oka S Clinical outcomes of endoscopic resection for superficial non-ampullary duodenal tumors Endosc Int Open 2020 8 E354 E359 10.1055/a-0998-3708 32118107 PMC7035028

[3] Tashima T Nonaka K Kurumi H Successful traction-assisted endoscopic submucosal dissection using dental floss and a clip for a huge superficial nonampullary duodenal epithelial tumor with severe fibrosis (with video) JGH Open 2019 3 179 181 31061895 10.1002/jgh3.12118 PMC6487824

